# A Mixed Methods Approach as a Channel to Interpret Outcomes Research and Lived Experience Enquiry of Upper Extremity Elective Surgery for Tetraplegia

**DOI:** 10.3390/jpm13030394

**Published:** 2023-02-23

**Authors:** K. Anne Sinnott Jerram, Jennifer Dunn, Richard Smaill, James Middleton

**Affiliations:** 1 John Walsh Centre for Rehabilitation Research, Kolling Institute, Royal North Shore Hospital, St Leonards, NSW 2065, Australia; 2 Faculty of Medicine and Health, Sydney Medical School-Northern, The University of Sydney, Camperdown, NSW 2006, Australia; 3 Burwood Academy Trust, Christchurch 8083, New Zealand; 4 Department of Orthopedic Surgery & Musculoskeletal Medicine, University of Otago, Christchurch 8011, New Zealand

**Keywords:** mixed methods, tetraplegia, upper extremity surgery, peers, cognitive demands

## Abstract

Cervical spinal cord injury (SCI) causing tetraplegia is extremely disabling. In such circumstances, restoration of upper extremity (UE) function is considered the highest priority. The advent of early nerve transfer (NT) procedures, in addition to more traditional tendon transfers (TT), warranted in-depth consideration given the time-limited nature of NT procedures. Potential surgery candidates may not yet have come to terms with the permanence of their disability. A mixed methods convergent design was utilized for concurrent analysis of the Aotearoa/New Zealand upper limb registry data from the clinical assessments of all individuals considering UE surgery, regardless of their final decision. The International Classification of Functioning, Disability and Health (ICF) taxonomy guided data interpretation during the three-phased study series. It was the integration of the findings using the Stewart Model of care drawn from palliative health that enabled the interpretation of higher order messages. It is clear the clinical assessment and selection processes in use require reconsideration given the complexities individuals face following onset of SCI. We draw attention to the higher order cognitive demands placed on individuals, the requirement for SCI peer involvement in decision making and the need for acknowledgment of interdependence as a relational construct when living with tetraplegia.

## 1. Introduction

Cervical spinal cord injury (SCI) causing tetraplegia involves damage to the central nervous system resulting in a variable loss of sensation, muscle paralysis and autonomic dysfunction below the level of injury [1]. The level and extent (severity) of neurological impairment determines the residual functional capacity and muscles that remain under voluntary control, with complete tetraplegia having the greatest degree of severity [2], and therefore, being the most disabling. Previous research has shown the improvements in upper extremity (UE) capacity may be enormously rewarding, useful and life enhancing [3,4,5]. Until recently, UE surgical reconstructions were restricted to the availability of innervated muscles for tendon transfer (TT) [6] and/or functional electrical stimulation (FES) [7], but the use of innovative nerve transfer (NT) surgeries has become more widespread [8,9]. Traditionally, both TT and FES reconstructive procedures are considered an option once an individual had reached a plateau of neurological recovery, which is usually at least 6–12 months following injury [9]. However, NT surgery needs to be performed early enough to allow muscle reinnervation before irreversible motor endplate degeneration occurs [10]. This means that ideally, NT surgery should be performed before 12 months post-injury but preferably earlier [11]. The time-limited nature of these new NT procedures, and the need for early ascertainment of the type of motor neuron lesion [12], has required that UE surgery is ideally performed within the first 6–9 months post-injury. This requirement raises new challenges for clinicians in relation to accuracy of prognostication, as well as for the individuals assessed as suitable for UE surgery. It is important to emphasize that the functional benefits of UE surgery for tetraplegia are not in question [13]. However, gaining more insight along with a fuller appreciation of the experience of the disability arising from tetraplegia is essential. Curiously, there is little reference to what is agreed to be ‘disablement’ [14,15] in the field of UE surgery research in this population. Typically, comprehensive rehabilitation following SCI is provided by an interdisciplinary specialist team and focuses on physical retraining and exercise, developing new procedural skills along with compensatory strategies and adaptive equipment. The goals of rehabilitation include independent living and self-management, as well as psychosocial support to assist adjustment and coping [16,17]. It is well established that hope is a priority and an important facilitator of adjustment following SCI [18]. Equally, hope provides a sense of inconsistency, contradiction (in terms of expectations of surgery) and even paradox [19]. The aim of this paper is to provide a deeper, enhanced appreciation of the life impacts of UE reconstructions. To do this, we describe a study series that discovered convergence between clinician-directed patient reported outcome measures (PROMs), a single clinical test and the voice from ‘lived-experience’ of tetraplegia on the topic of elective UE surgery. Each study was designed to complement and build upon one another, more so than conducting a qualitative or quantitative investigation in isolation. This paper reports the data convergence process and results using a complementary method of analysis.

## 2. Materials and Methods

**Figure 1:**
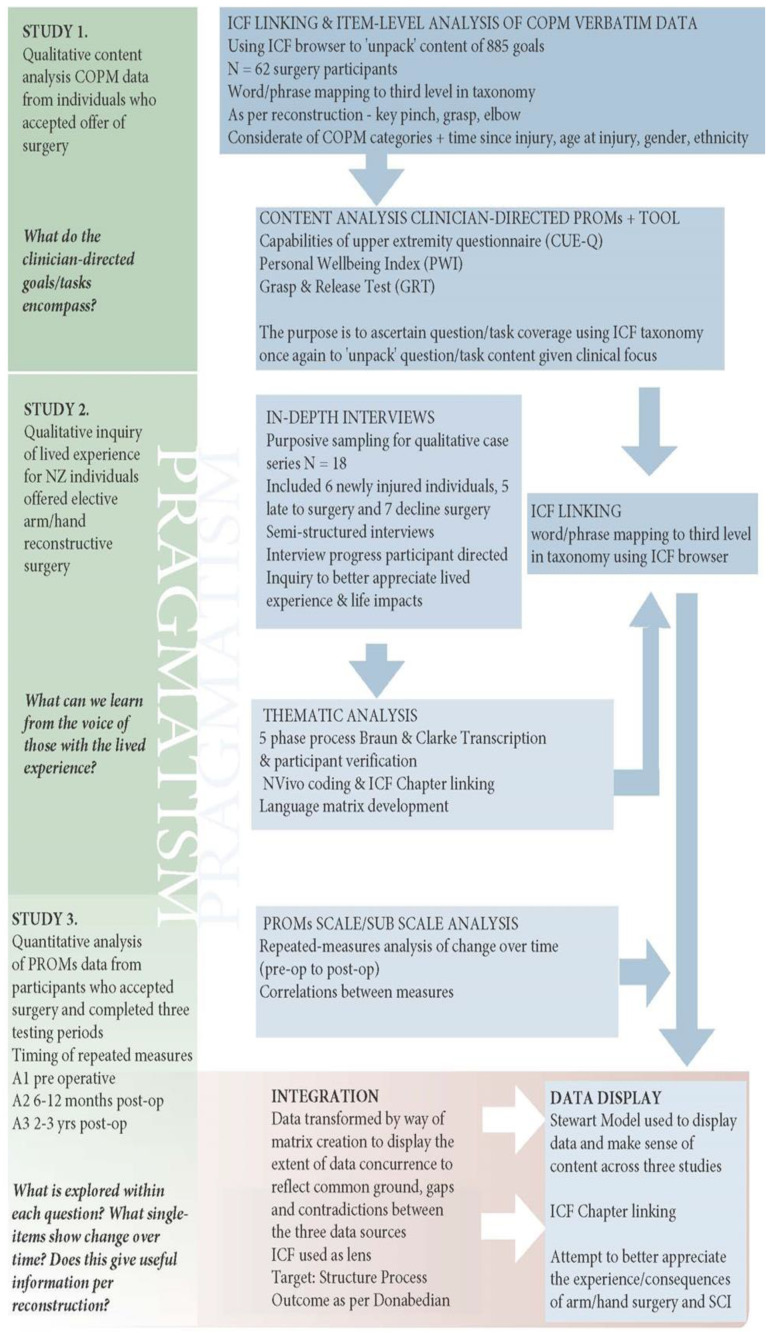
Overall study proposal schema.

We used a mixed methods convergent design comprising two qualitative studies and one quantitative study. A schema for the overall design is presented in Figure 1. The International Classification of Functioning, Disability and Health (ICF) [20], taxonomy was used as the analytical lens to guide data interpretation, as per international therapist consensus [21]. The requirement for a pragmatic focus on study design, data collection and data analysis were emphasized and reflected in the concurrent design. All phases of the study series were granted equal priority, based on the rationale that while independently they addressed specific research objectives, all three studies were integral to realizing the overall research question. 

The study protocol [22], as well as methods and results of the content analysis of clinical outcome measures [23] and analysis of clinical outcomes data [24], have been published previously. To assist understanding, the key steps are summarized below. 

Study 1: Content analysis of three patient-reported outcome measures and a single timed-test.

The first study described the extent of coverage of human functioning for the four tools by linking the content of each question in the clinical outcome measures to the ICF [24]. The tools included three PROMS, the Canadian Occupational Performance Measure (COPM) [25], Capabilities of Upper Extremity questionnaire (CUE-Q) [26] and the Personal Wellbeing Index (PWI) [27]. The single clinical timed test was the Grasp and Release Test (GRT) [28]. A word-by-word content analysis of each question/activity was performed and linked to the ICF taxonomy using specific linking rules [29]. 

Study 2: In-depth Interviews.

The second study was a qualitative case series exploring the lived experience at various time points between the onset of tetraplegia and decision about whether to either accept or decline arm/hand reconstruction surgeries. This included early NT and TT procedures and individuals who had declined surgery. The aims of the study were to better appreciate what individuals identify, experience and perceive to be the influences determining the choice of UE surgery. All participants were provided with an information sheet and signed a consent form prior to interview. In-depth semi-structured interviews were conducted, and transcripts analyzed using reflexive thematic narrative analysis [30,31]. Recruitment was purposive to ensure broad representation in terms of the lived-experience response to the offer of elective arm/hand surgery. Individuals who had elected to have UE surgery many years following SCI and those who had declined surgery were specifically targeted. All participants in both the late-to-surgery group and the declined-to-have surgery group were explicitly asked about what their advice would be based on current knowledge and life experience to: (i) a newly injured person in the spinal cord injuries unit today and (ii) their younger self. Data were managed using the qualitative data analysis software NVivo (Version 12: QSR International Pty Ltd., Burlington, MA, USA). 

Study 3: Analysis of clinical outcomes data.

The third study examined changes in pre- and post-surgery scores for the three PROMs (COPM, CUE-Q and PWI) and the GRT [23]. This study had two aims. First, to assess single item sensitivity to change based on groupings of surgical reconstructions that best represent the participants with differing SCI severity. Second, to analyze pre- to post-surgery changes in total summed scores and assess change over time for prioritized goals, self-reported UE task-based capacity, self-reported perception of well-being and grasp and release arm/hand ability. Participants were grouped according to their SCI level and resultant surgical procedures into higher SCI severity and lower SCI severity groups. It is routine clinical practice at the Burwood Spinal Unit for bilateral and simultaneous surgery, where firstly elbow reconstruction and then pinch and/or grip reconstruction is performed. All NT procedures were the nerve to supinator being transferred to the posterior interosseous nerve (SPIN) and were performed at least six months prior to any TT procedures. 

### Data Integration

**Figure 2:**
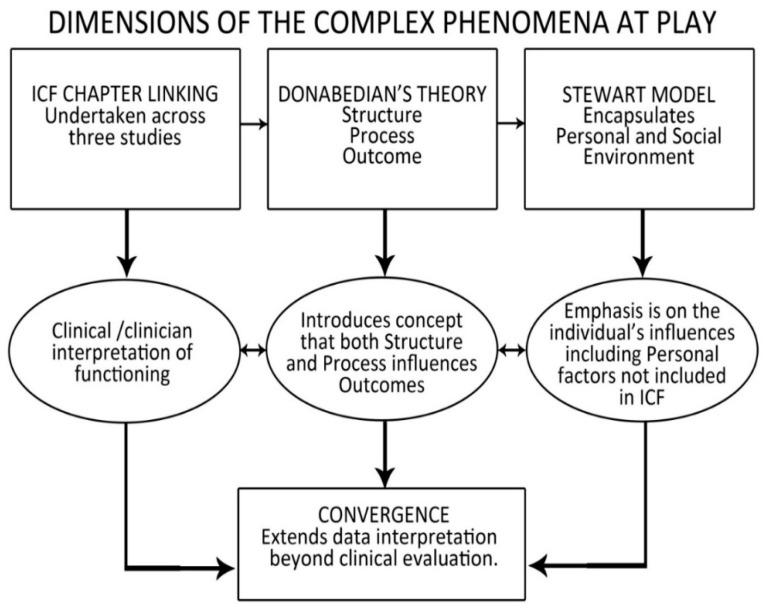
Data integration process-seeking ‘complementarity’.

The objective of this mixed methods design was to explore different aspects or ‘dimensions’ of the same complex phenomenon of interest [32]. The data integration process is represented by a flow diagram in Figure 2. The linking to the ICF taxonomy provided the analytical lens and underpins the coherence, to enable the integration of data from the three studies. For example, linking the goal/task data from Study 1 to the ICF taxonomy and linking the in-depth interview codes from Study 2 to ICF categories to the same taxonomy created the possibility to look for conflicting data, with inconsistency or gaps. This process provided the opportunity for both content analysis and frequency of ICF linkages from both sets of data, while also comparing the ICF linkages to the content of the questions, in terms of scope of human function and contextual factors. (Refer to ICF linking in Supplementary Material in Table S1). The second step to integration of the results of the three studies was to systematically divide the data sources according to Donabedian’s quality and safety framework [33] to provide a Donabedian styled roadmap based on structure, process and outcome. The justification for this division of data sources across Structure, Process and Outcome is that Donabedian’s framework provides a “step-wise roadmap to guide healthcare systems and organizations in the provision of person-centered care” [34] (p. 429). Lastly, we chose the Stewart Model [35] drawn from Donabedian’s framework to provide a broader ‘macro’ picture of life impacts by drawing immediate attention to the Personal and Social Environment prior to the consideration of Donabedian’s domains. Notably, the Personal Factors domain is currently missing from the ICF taxonomy [36,37]. 

## 3. Results

The key findings from each study are separately summarized in Table 1. Study 1 suggests the priorities of individuals may change at different points in time after injury. It found that the ‘pursuit of independence’ was greatest for newly injured individuals who have limited experience of life with tetraplegia. In addition, there was a greater emphasis on the self-care category for those with more experience of living with SCI versus the greater emphasis on leisure from newly injured individuals. This may reflect different values across the lifespan, although the age ranges were comparable. A broader perspective on human functioning was provided from the prioritized COPM tasks than any of the clinical outcome tools used in terms of scope of questions. Study 2 drew attention to the possibility that UE surgery is more cognitively demanding than has previously been reported. Findings demonstrated that individuals were overwhelmed by post-acute rehabilitation requirements and emphasized the enormity of dealing with SCI, which took priority over UE function, despite the obvious impairments. Late-to-surgery participants provided further insight in relation to functional priorities with bladder and bowel being ranked the highest, as well as the place of ‘hope for better than now’ and how this influences decision making, along with the clinical selection and assessment processes. Peers with SCI are enormously influential in terms of decision-making processes and are under-utilized in hand clinic service delivery. Study 3 provides new information on the use of CUE-Q and PWI as PROMs in this population. Results showed favorable changes at 6–12 months post-surgery for all groups. In addition, pre- to post-op score changes highlight the differences in observed functional task completion and perceived capacity. The poor correlations between certain measures draws attention to the discrepancies between changes in function and changes in perceived wellbeing in addition to the influence of individuals’ expectations.

### Integration of Results

**Figure 3:**
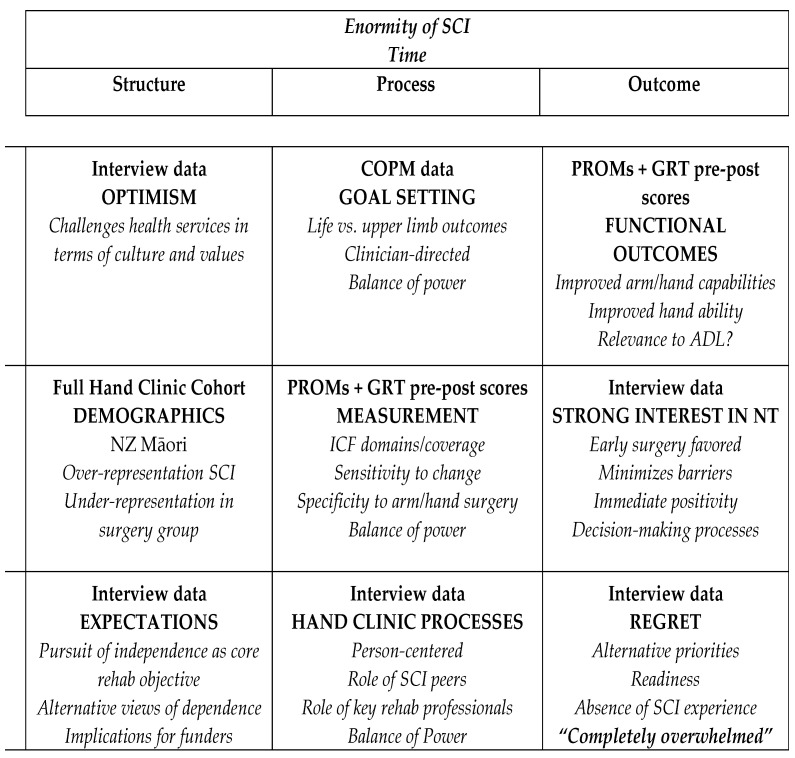
Donabedian’s road map in relation to structure, process and outcome.

The first data integration step was to figuratively represent the ICF chapter coverage across the data sources from the three studies and the results have been reported [24]. Notably, while mobility, self-care and environmental influences dominated, it was only through the interview narrative data that the extent of Mental Function demands were revealed. The ICF interpretation of mental functions includes orientation functions, emotional functions, including optimism and motivation, and higher-level cognitive functions. The second data integration step was to divide the results from the three studies into Donabedian’s framework under the headings structure, process and outcomes. With reference to structure, Study 2 interview data revealed optimism as a challenge to health services in terms of culture and values and challenged the pursuit of independence as the core objective of rehabilitation. Study 3 revealed an over representation of indigenous Māori in the group who declined to have surgery. With reference to process, Study 1 drew attention to broader life versus UE surgery goals, emphasized the clinician-directed interview mode and resultant balance of power issues. Study 2 challenged the notion of person-centeredness, drew attention to the role of peers with SCI and key rehabilitation professionals, as well as balance of power issues, while Study 3 drew attention to the tools themselves in terms of the ICF domains/coverage, their sensitivity to change, specificity to UE surgery and suitability of tasks tested. With respect to outcomes, Study 1 emphasized the strong interest in the recent NT innovations seen as immediate positivity, alongside Study 2′s revelations around readiness, absence of SCI experience and the regret in terms of the decision to decline surgery. Study 3 drew attention to the differences in improved UE capability and changes in function in terms of the relevance to activities of daily living (ADL). The macro, meso and micro level of this systematic division is displayed Figure 3.

Sitting above “structure, process and outcome” is ‘the enormity of SCI’ and ‘time since injury’, which are two elements that influence each of the three domains. This is irrespective of age at time of SCI, which allowed for the consideration of differences in values across life span. 

**Figure 4:**
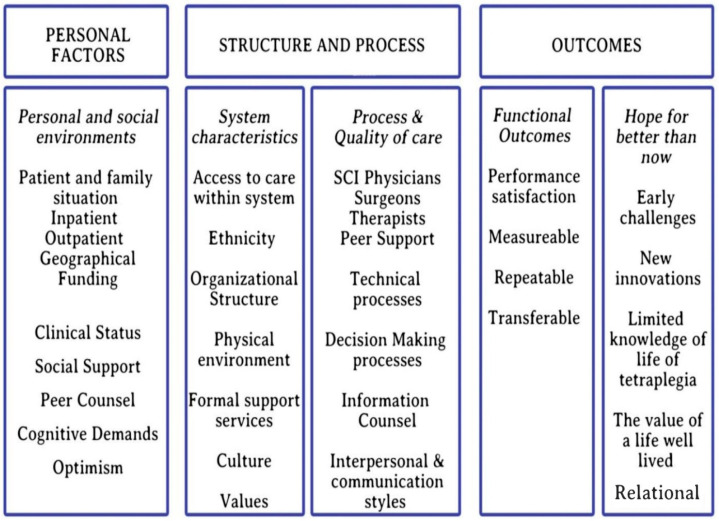
Conceptual framework for interpretation of results based on the Stewart Model.

The third step was the clinical interpretation using the Stewart Model shown in Figure 4. In the first instance, the individual and their Personal and Social Environment are considered to reflect voice from the ‘lived experience’ dimension of this study series. Elements included patient and family situation, inpatient vs. outpatient status, geographical location, funding, clinical status, social support, peer counsel, cognitive demands and optimism. Second, structure and process are looked at from a SCI rehabilitation procedural perspective. A division into system characteristics included access to care within the system, ethnicity, organizational structure, physical environment, formal support services, culture and values. The process and quality of care category included specialists/clinicians, peers with SCI, technical processes, decision-making processes, information/counsel and interpersonal communications styles. Lastly, outcomes were considered as either functional outcomes or hope for better than now. The elements of the former included aspects of performance and satisfaction, and being measurable, repeatable and transferable. The elements of the latter included early challenges, new innovations, limited knowledge of life with tetraplegia, the value of a life well lived and the relational characteristics of all elements. 

## 4. Discussion

A mixed methods approach allowed for greater appreciation of the relational improvement of UE function in terms of life with tetraplegia. We signal that in the presence of acute tetraplegia, there is a varied human response to elective UE requirements and there is much to consider. Firstly, while this analysis draws attention to the central issue of timing, debate in the rehabilitation literature about suitable processes and frameworks for goal setting highlights that there are other important issues to address than simply timing [38]. The controversy as to whether goal pursuits must be autonomous or integrated to the self is of interest and may explain why, in Study 1 the newly injured individuals’ COPM goals were more focused on the future, less specific and took a broader view of what they hoped the results of surgery might achieve [23]. These participants had little or no ability to truly envisage a life with the extent of paralysis their spinal injury accorded each of them. Secondly, emphasis on the scale of the life demands of tetraplegia and the “magnitude of my SCI” captured by the in-depth interviews in Study 2 raises the value of ‘life goals’ as a core conversation either prior to or within the hand clinic setting. Consideration of life goals is important in rehabilitation service delivery because of their influence over client motivation and wellbeing, yet the primacy of the individuals’ perspective in this field has never been verified [39]. This is an example of the higher-level cognitive demands that are significant for these individuals while completing post-acute rehabilitation and coming to terms with an extraordinary level of physical impairment. Cognitive impairment following acute SCI related to a sense of being overwhelmed/anxious, poor sleep quality and/or pharmacology has recently been highlighted [40]. Next, the results of Study 3 draw attention to the differences in measurement of UE task performance (as measured by the GRT) and capacity (as reported by the CUE-Q), while at the same time, capturing the PWI results suggest that there are important changes in perception of three dimensions of personal wellbeing not previously reported in the UE surgery literature [23]. These include the person and their family situation and social geography. This is paramount because individual’s expectations need to be considered [9], especially when newly injured individuals are invited to undergo a full clinical assessment and process of informed consent for a time-limited intervention in the post-acute rehabilitation setting.

Finally, in terms of clinicians, highly specialized UE surgery teams have the skills, the mandate, and the power to improve the person-centeredness of their assessment, measurement and goal setting processes. It behooves them to ensure the choice around UE surgery reconstructions is better informed, truly shared in terms of decision making and mindful of the likely absence of any real understanding of the life with disability that awaits beyond the hospital doors. It was clear from this analysis that peers with SCI are enormously influential in terms of assisting in decision-making processes and are under-utilized in hand clinic service delivery. This finding has been found in previous studies in this population [41]. Individuals with tetraplegia need to be referred to these services to be fully informed about their options, and the service needs to be accessible/flexible to meet their needs. Significant differences in access and equity to such services are apparent within the literature for UE surgery for tetraplegia, where in the United States, only 7% of eligible people with tetraplegia have UE reconstructive procedures [42,43,44] compared to Aotearoa/New Zealand, with the uptake closer to half the eligible population [45]. However, urgent investigation is required given previously reported differences for surgery uptake for Māori in this cohort [23]. With new innovations emerging, the recommendation is for clinicians to proceed with caution and remain mindful of cultural disparities, competing interests and daily challenges for newly injured individuals with tetraplegia, who quite possibly have alternative priorities that may push the limits of clinicians’ perceptions of normality. By synthesizing the data generated from the three studies, we have presented a more refined consideration of the disablement of tetraplegia while navigating UE elective surgery. 

## 5. Conclusions

We draw attention to the higher-order cognitive demands placed on individuals, the requirement for involvement of peers with SCI in decision making and the need for acknowledgment of interdependence as a relational construct when living with tetraplegia. Responsibility, care and interdependence, and their relationships, are the key attributes required to develop a sense of self and form capacities and life plans for the life that awaits with tetraplegia. We argue it is the relational aspects of UE surgery and life with tetraplegia that requires further investigation to enhance shared care planning and service delivery.

## Figures and Tables

**Table 1:** Main findings from the three studies.

	FOCUS/ DESIGN	METHODS	SUMMARY OF KEY FINDINGS
**STUDY 1** Qualitative content analysis COPM data from individuals who accepted the offer of surgery	Word mapping exercise using the ICF taxonomy	Sample includes all individuals identified as suitable for surgery (n = 59). Five tasks identified per reconstruction in the domains of *self-care, productivity and leisure*. Followed by single question content analysis of all tools used in Hand Clinics in NZ. Word/phrase mapping to third level ICF taxonomy per reconstruction—key pinch, grasp, elbow with or without NT.	(i) Evidence that there are differences in the priorities of individuals across the three COPM domains at <1 year and >10 years post SCI, irrespective of age at time of SCI. (ii) Greater emphasis on the ‘pursuit of independence’ for newly injured individuals who have limited experience of life with tetraplegia. (iii) The greater emphasis on the self-care category for those with more experience of SCI vs. the greater emphasis on leisure from newly injured individuals draws attention to the crucial role of the clinician directing the interview. This may reflect different values across the lifespan, although the age ranges were comparable. A broader perspective on human functioning was provided from the prioritised COPM tasks than any of the clinical outcome tools used in terms of scope of questions.
**STUDY 2** Qualitative inquiry of lived experience for individuals offer arm/hand surgery	In-depth interviews	Semi-structured interviews of early NT surgery recipients (n = 6), late TT surgery recipients (n = 5) and those who declined to have reconstructive surgery (n = 7).	(i) Evidence that the requirement for UE surgery is more cognitively demanding than previously reported, that individuals are overwhelmed by post-acute rehabilitation requirements and emphasizes the enormity of SCI, which takes priority over UE function, despite the obvious impairments. (ii) Late to surgery participants provided clarity in terms of functional priorities with bladder and bowel highest. (iii) The place of ‘hope for better than now’ influences decision-making, as well as the clinical selection and assessment processes. (iv) SCI peers are enormously influential in terms of decision-making processes and under-utilized in hand clinic service delivery.
**STUDY 3** Quantitative analysis of PROMs data	Descriptive-correlational analysis	Analysis of pre-and post-operative scores of the COPM, CUE-Q and PWI data from participants and the GRT (in select cases) who accepted surgery and completed three testing periods.	(i) Provides new information on the use of CUE-Q and PWI as PROMs in this population, showing favorable changes at 6–12 months post-surgery for all groups. (ii) Results highlight the differences in observed functional task completion and perceived capacity. (iii) Poor correlations between measures draws attention to the discrepancies between changes in function and changes in perceived wellbeing, in addition to the influence of individuals’ expectations.

## Data Availability

All data are available as Supplementary Material.

## Supplementary Materials

The following supporting information can be downloaded at: https://www.mdpi.com/article/10.3390/jpm13030394/s1, Table S1: Data Source and ICF linking to 2nd (or 3rd level coding in brackets if this detail provides clarity).
