# Supplementary material for: A Mixed Methods Approach as a Channel to Interpret Outcomes Research and Lived Experience Enquiry of Upper Extremity Elective Surgery for Tetraplegia
Source: J Pers Med. 2023 Feb 23;13(3):394. doi: 10.3390/jpm13030394 (PMC10058672; doi:10.3390/jpm13030394)
Supplement: Supplementary file 1 [file jpm-13-00394-s001.zip › jpm-2119435-supplementary.pdf]

Supplementary Material: Data Source and ICF linking to 2nd (or 3rd level coding in brackets if this detail provided clarity)

Table S1. Data Source and ICF linking to 2nd (or 3rd level coding in brackets if this detail provided clarity)

Data Source	Body Structures (s)	Body Functions (b)	Activity & Participation (d)	Environmental Factors (e)
**Subordinate themes**				
Triumphs of hope over inexperience of SCI		**Chapter 1: Mental Functions**<br>b114 Orientation Functions<br>b152 Emotional functions<br>(b1265 Optimism)<br>b164 Higher-level cognitive functions	**Chapter 1: Learning and applying knowledge**<br>d130-159 Basic learning<br>d155 Acquiring skills<br>**Chapter 5 Self-care**<br>d570 Looking after one's health<br>(d5701 Managing diet and fitness)<br>**Chapter 7: Community, social and civic life**<br>d720 Complex interpersonal interactions<br>**Chapter 8 Major life areas**<br>d870 Economic self-sufficiency	**Chapter 4: Attitudes**<br>e460 Societal attitudes
Deal breakers included driving		**Chapter 1: Mental Functions**<br>b114 Orientation Functions<br>b152 Emotional functions<br>(b1301 Motivation)<br>b164 Higher-level cognitive functions	**Chapter 1: Learning and applying knowledge**<br>d155 Acquiring skills d130-159 Basic learning<br>**Chapter 5 Self-care**<br>(d5700 Ensuring one's physical comfort)<br>**Chapter 8 Major life areas**<br>d870 Economic self-sufficiency	**Chapter 4: Attitudes**<br>e460 Societal attitudes

Driving	**Chapter 7: Structures related to movt.**<br>s720 Structure of shoulder region	**Chapter 4: Functions of the cardiovascular system**<br>(b4550 General physical endurance)	**Chapter 4 Mobility**<br>d440 Fine hand use<br>(d4401 Grasping)<br>(d4402 Manipulating)<br>(d4452 Reaching)<br>(d4453 Turning or twisting the hands or arms)<br>d429 Changing and maintaining body position<br><br>**Chapter 5 Self-care**<br>d570 Looking after one's health<br>d920 Recreation and leisure	**Chapter 5: Services, systems & policies**<br>e540 Transportation services, systems and policies
Don't underthink the future		**Chapter 1: Mental Functions**<br>b114 Orientation Functions<br>(b1265 Optimism)<br>b152 Emotional functions<br>b164 Higher-level cognitive functions	**Chapter 1: Learning and applying knowledge**<br>d155 Acquiring skills d130-159 Basic learning	**Chapter 4: Attitudes**<br>e460 Societal attitudes
Nothing to lose		**Chapter 1: Mental Functions**<br>b110 Consciousness<br>b1265 Optimism<br>b130 Energy and drive functions<br>(b1301 Motivation)<br>b152 Emotional functions<br>b164 Higher-level cognitive functions	**Chapter 1: Learning and applying knowledge**<br>d130-159 Basic learning	**Chapter 4: Attitudes**<br>e460 Societal attitudes
Don't be rushed		**Chapter 1: Mental Functions**	**Chapter 5 Self-care**<br>d570 Looking after one's health	**Chapter 3 Relationships**<br>e310 Immediate family<br>e315 Extended family

		b130 Energy and drive functions<br>b152 Emotional functions<br>b164 Higher-level cognitive functions		e320 Friends<br>e325 Acquaintances, peers, colleagues, neighbours and community members<br>**Chapter 4: Attitudes**<br>e425 Individual attitudes of acquaintances, peers, colleagues, neighbours and community members<br>e450 Individual attitudes of health professionals<br>e460 Societal attitudes
SCI peers do know		**Chapter 1: Mental Functions**<br>b152 Emotional functions<br>b164 Higher-level cognitive functions		**Chapter 4: Attitudes**<br>e425 Individual attitudes of acquaintances, peers, colleagues, neighbours and community members
Yet we are all different		**Chapter 1: Mental Functions**<br>b110 Consciousness<br>b152 Emotional functions<br>b164 Higher-level cognitive functions		**Chapter 4: Attitudes**<br>e425 Individual attitudes of acquaintances, peers, colleagues, neighbours and community members
Not ready to see self in peers		**Chapter 1: Mental Functions**<br>b152 Emotional functions<br>b164 Higher-level cognitive functions	**Chapter 7 Interpersonal interactions and relationships**<br>d720 Complex interpersonal interactions	
Clinicians know but don't know		**Chapter 1: Mental Functions**<br>b152 Emotional functions<br>b164 Higher-level cognitive functions	**Chapter 7 Interpersonal interactions and relationships**<br>d720 Complex interpersonal interactions	**Chapter 3 Relationships**<br>e355 Health professionals<br>**Chapter 4: Attitudes**<br>e450 Individual attitudes of health professionals
Always hoping for better		**Chapter 1: Mental Functions**<br>(b1265 Optimism)		

		b164 Higher-level cognitive functions		
Hope for recovery		**Chapter 1: Mental Functions**<br>b110 Consciousness<br>b152 Emotional functions<br>b164 Higher-level cognitive functions	**Chapter 5 Self-care**<br>d570 Looking after one's health	**Chapter 3 Relationships**<br>e355 Health professionals<br>**Chapter 4: Attitudes**<br>e450 Individual attitudes of health professionals
Regret re timing		**Chapter 1: Mental Functions**<br>b164 Higher-level cognitive functions		**Chapter 4: Attitudes**<br>e430 Individual attitudes of people in positions of authority
Funding discrepancies		**Chapter 1: Mental Functions**<br>b164 Higher-level cognitive functions		**Chapter 5: Services, systems & policies**<br>e565 Economic services, systems, and policies<br>e570 Social security services, systems and policies<br>e580 Health services, systems, and policies
Social geography		**Chapter 1: Mental Functions**<br>b164 Higher-level cognitive functions	**Chapter 7 Interpersonal interactions and relationships**<br>d720 Complex interpersonal interactions	**Chapter 4: Attitudes**<br>e460 Societal attitudes<br>**Chapter 5: Services, systems & policies**<br>e525 Housing services, systems, and policies<br>e565 Economic services, systems, and policies<br>e570 Social security services, systems and policies
Power of case managers		**Chapter 1: Mental Functions**<br>b164 Higher-level cognitive functions	**Chapter 7: Community, social and civic life**<br>d720 Complex interpersonal interactions	**Chapter 4: Attitudes**<br>e430 Individual attitudes of people in positions of authority
Acceptance vs. adjustment		**Chapter 1: Mental Functions**<br>b110 Consciousness<br>b152 Emotional functions<br>b164 Higher-level cognitive functions		**Chapter 3 Relationships**<br>e310 Immediate family<br>e315 Extended family<br>e320 Friends<br>e325 Acquaintances, peers, colleagues, neighbours, and community members<br>**Chapter 4: Attitudes**<br>e460 Societal attitudes<br>**Chapter 5: Services, systems & policies**

				e525 Housing services, systems, and policies<br>e515 Architecture and construction services, systems, and policies<br>e575 General social support services, systems, and policies
**COPM priorities**				
	**Chapter 5: Structures related to the digestive, metabolic and endocrine systems**<br>540 Structure of the intestine	**Chapter 2: Sensory functions and pain**<br>b270 Sensory functions related to temperature	**Chapter 1: Learning and applying knowledge**<br>d155 Acquiring skills	**Chapter 5: Services, systems & policies**<br>e110 Products or substances for personal consumption<br>e115 Products and technology for personal use in daily living<br>e120 Products and technology for personal indoor and outdoor mobility and transportation<br>e125 Products and technology for communication<br>e130 Products and technology for education<br>e140 Products and technology for culture, recreation, and sport<br>e150 Design, construction and building products and technology of buildings for public use<br>e155 Design, construction and building products and technology of buildings for private use
	**Chapter 6: Structure related to genitourinary and reproductive systems**<br>s610 Structure of urinary system	**Chapter 4: Functions of the cardiovascular system**<br>b455 Exercise tolerance functions	**Chapter 1: Learning and applying knowledge**<br>d170 Writing	**Chapter 2: Natural environment and human-made changes**<br>e210 Physical geography<br>e225 Climate
	**Chapter 7: Structure related to movement**<br>s710 Structure of head and neck region<br>s720 Structure of shoulder region<br>s730 Structure of upper extremity<br>s740 Structure of pelvic region	**Chapter 6: Function related to genitourinary and reproductive systems**<br>b610 Urinary excretory functions	**Chapter 2: General tasks and demands**<br>d210 Undertaking a single task<br>d230 Carrying out daily routine	**Chapter 3: Support and relationships**<br>e310 Immediate family<br>e315 Extended family<br>e320 Friends<br>e325 Acquaintances, peers, colleagues, neighbours, and community members<br>e340 Personal care providers and personal assistants<br>e350 Domesticated animals<br>e355 Health professionals

	s750 Structure of lower extremity<br>s760 Structure of trunk<br>s760 Structure of trunk<br>s810 Structure of areas of skin			
		**Chapter 7: Function related to movement**<br>b710 Mobility of joint functions<br>b715 Stability of joint functions<br>b730 Muscle power functions<br>b740 Muscle endurance functions	**Chapter 3: Communication**<br>d360 Using communication devices and techniques	**Chapter 4: Attitudes**<br>e410 Individual attitudes of immediate family members<br>e420 Individual attitudes of friends<br>e425 Individual attitudes of acquaintances, peers, colleagues, neighbours, and community members<br>e430 Individual attitudes of people in positions of authority<br>e445 Individual attitudes of strangers<br>e450 Individual attitudes of health professionals<br>e455 Individual attitudes of health-related professionals<br>e460 Societal attitudes
			**Chapter 4: Mobility**<br>d410 Changing basic body position<br>d415 Maintaining a body position<br>d420 Transferring oneself<br>d430 Lifting and carrying objects<br>d440 Fine hand use<br>d460 Moving around in different locations<br>d465 Moving around using equipment<br>d470 Using transportation<br>d475 Driving	
			**Chapter 5: Self-care**<br>d510 Washing oneself<br>d520 Caring for body parts<br>d530 Toileting<br>d540 Dressing<br>d550 Eating	**Chapter 5: Services, systems, and policies**<br>e510 Services, systems, and policies for the production of consumer goods<br>e515 Architecture and construction services, systems, and policies<br>e525 Housing services, systems, and policies

			d560 Drinking<br>d570 Looking after one's health	e535 Communication services, systems, and policies<br>e540 Transportation services, systems, and policies<br>e560 Media services, systems, and policies<br>e565 Economic services, systems, and policies<br>e570 Social security services, systems and policies<br>e575 General social support services, systems, and policies<br>e580 Health services, systems, and policies
			**Chapter 6: Domestic life**<br>d630 Preparing meals<br>d640 Doing housework<br>d650 Caring for household objects<br>d660 Assisting others	
		.	**Chapter 7: Interpersonal interactions and relationships**<br>d720 Complex interpersonal interactions<br>d760 Family relationships<br>d770 Intimate relationships	
			**Chapter 8: Major life events**<br>d825 Vocational training<br>d830 Higher education<br>d845 Acquiring, keeping and terminating a job<br>d850 Remunerative employment<br>d855 Non-remunerative employment<br>d870 Economic self-sufficiency	
			**Chapter 9: Community, social and civic life**<br>d910 Community life<br>d920 Recreation and leisure	
**CUE-Q tasks**				
	**Chapter 7: Structure related to movement**	**Chapter 7: Function related to movement**	**Chapter 3: Communication**<br>d360 Using telecommunication devices	

	s710 Structure of head and neck region<br>s720 Structure of shoulder region<br>s730 Structure of upper extremity<br>s760 Structure of trunk	b710 Mobility of joint functions<br>b715 Stability of joint functions<br>b720 Mobility of bone functions<br>b7200 Mobility of scapula<br>b7202 Mobility of carpal bones<br>b730 Muscle power functions<br>(b7300 Power of isolated muscles/groups)		
			**Chapter 4: Mobility**<br>d440 Fine hand use<br>(d4452 Reaching)<br>(d4400 Picking up)<br>(d4401 Grasping)<br>(d4402 Manipulating)<br>(d4453 Turning or twisting the hands or arms)<br>(d4300 Lifting)<br>d429 Changing and maintaining body position	
			**Chapter 5: Self-care**<br>d520 Caring for body parts<br>(d5200 Caring for skin)<br>(d5201 Caring for teeth)<br>d550 Eating<br>d560 Drinking<br>d570 Looking after one's health	
			**Chapter 6: Domestic life**<br>d650 Caring for household objects	
			**Chapter 8: Major life events**<br>d860 Basic economic transactions	

**PWI question content**				
			**Chapter 5: Self-care**<br>d570 Looking after one's health	**Chapter 4: Attitudes**<br>e460 Societal attitudes
			**Chapter 7: Interpersonal interactions and relationships**<br>d710 Basic interpersonal interactions<br>d720 Complex interpersonal interactions<br>d740 Formal relationships<br>d750 Informal social relationships<br>d760 Family relationships<br>d770 Intimate relationships	**Chapter 5: Services, systems, and policies**<br>e515 Architecture and construction services, systems/policies<br>e525 Housing services, systems, and policies<br>e530 Utilities services, systems, and policies<br>e565 Economic services, systems, and policies<br>e570 Social security services, systems and policies<br>e580 Health services, systems, and policies
			**Chapter 8: Major life events**<br>d870 Economic self-sufficiency	
			**Chapter 9: Community, social and civic life**<br>d910 Community life<br>d920 Recreation and leisure<br>d930 Religion and spirituality	
**GRT tasks**				
	**Chapter 7: Structure related to movement**<br>s710 Structure of head and neck region<br>s720 Structure of shoulder region<br>s730 Structure of upper extremity<br>s760 Structure of trunk	**Chapter 7: Function related to movement**<br>b710 Mobility of joint functions<br>b715 Stability of joint functions<br>b720 Mobility of bone functions<br>(b7200 Mobility of scapula)<br>(b7202 Mobility of carpal bones)<br>b730 Muscle power functions	**Chapter 4: Mobility**<br>d440 Fine hand use<br>(d4452 Reaching)<br>(d4400 Picking up)<br>(d4401 Grasping)<br>(d4402 Manipulating)<br>(d4453 Turning or twisting the hands or arms)<br>(d4300 Lifting)<br>d429 Changing and maintaining body position	

		b7300 Power of isolated muscles/groups		
